# Tunable Plasmonic Band-Pass Filter with Dual Side-Coupled Circular Ring Resonators

**DOI:** 10.3390/s17030585

**Published:** 2017-03-13

**Authors:** Dongdong Liu, Jicheng Wang, Feng Zhang, Yuewu Pan, Jian Lu, Xiaowu Ni

**Affiliations:** 1 School of Science, Nanjing University of Science & Technology, Nanjing 210094, China; gnod6@sohu.com (D.L.); jsnjnxw@gmail.com (X.N.); 2 School of Mathematics & Physics Science, Xuzhou University of Technology, Xuzhou 221018, China; yuewupan@163.com; 3 School of Science, Jiangsu Provincial Research Center of Light Industrial Optoelectronic Engineering and Technology, Jiangnan University, Wuxi 214122, China; 4 Key Laboratory of Semiconductor Materials Science, Institute of Semiconductors, Chinese Academy of Sciences, Beijing 100083, China; fzhang@semi.ac.cn

**Keywords:** integrated optics devices, waveguides, plasmonics, metal-insulator-metal (MIM), sensors

## Abstract

A wavelength band-pass filter with asymmetric dual circular ring resonators in a metal-insulator-metal (MIM) structure is proposed and numerically simulated. For the interaction of the local discrete state and the continuous spectrum caused by the side-coupled resonators and the baffle, respectively, the transmission spectrum exhibits a sharp and asymmetric profile. By adjusting the radius and material imbedded in one ring cavity, the off-to-on plasmon-induced absorption (PIA) optical response can be tunable achieved. In addition, the structure can be easily extended to other similar compact structures to realize the filtering task. Our structures have important potential applications for filters and sensors at visible and near-infrared regions.

## 1. Introduction

Surface plasmon polaritons (SPPs), a kind of transverse electromagnetic wave tightly confined at the interface between metal and dielectric material, is able to propagate up to a few micrometers [1,2]. Their outstanding ability for overcoming the classical optical diffraction limit has made SPPs attractive as energy and information carriers in highly integrated optical circuits and devices [3,4].

Among various SPP structures, insulator-metal-insulator (IMI) structures and metal-insulator-metal (MIM) structures are two important multilayer plasmonic structures. Due to not only supporting modes with deep sub-wavelength scales and high group velocity over a very wide range of frequencies but also offering very high optical confinement and acceptable propagation length [5], metal-insulator-metal (MIM) structures, such as optical filters [6,7,8,9], optical switches [10], demultiplexers [11,12], and sensors [13,14,15,16], are widely used. Plasmonic filters based on MIM waveguide structures, such as asymmetric nanodisk filter and sensor [17,18,19], side-coupled cavity sensor [20], notch resonator filter and sensor [21], and circular ring filter and sensor [22,23], are one of the most important optical devices, have attracted tremendous attention, and have been investigated widely in recent years. All of the above-mentioned devices are promising candidates for highly integrated optical circuits. Electromagnetically induced transparency (EIT), a quantum mechanical phenomenon, reduces light absorption over a narrow spectral region in a coherently driven atomic system [24]. However, hard operation conditions limit its development, and then alternative plasmon-induced transparency (PIT) effects that are analogous to the EIT effects, are demonstrated in various MIM waveguide structures [25,26,27]. Recently, plasmon-induced absorption (PIA), an opposite effect of the PIT effect, have been widely studied and demonstrated in the MIM structures [28,29,30]. Especially, PIA response is investigated in a MIM-based end-coupled composite-slot-cavity resonator [31]. These MIM structures will be beneficial for optical switching in highly integrated photonic devices.

In this paper, a wavelength filter consisting of dual side-coupled circular ring resonators and two waveguides with a baffle is proposed. Numerical simulation by finite element method (FEM) was conducted to analyze our designs. The results show PIA transmission in spectra. By changing the radius or the refractive index of one ring, we achieve an on-to-off PIA optical response. Moreover, similar structures were designed by moving the location of one ring of the above system. The corresponding transmission spectra and the magnetic intensity distributions |*H*_z_|^2^ at special wavelengths were investigated.

## 2. Model and Theoretical Analysis

**Figure 1:**
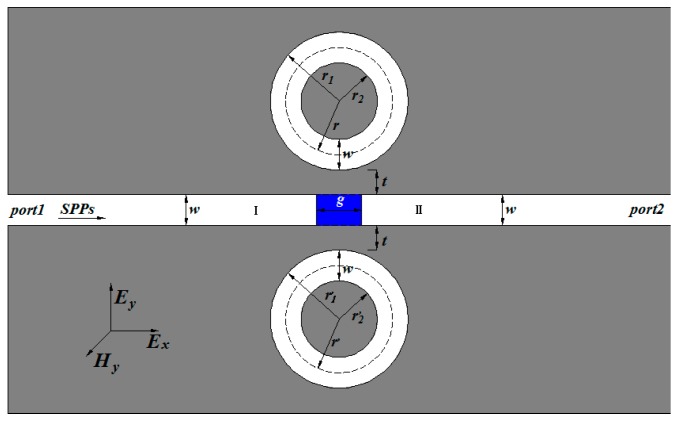
Schematic of the structure composed of two shunt-wound rings resonators with two waveguides and the structure parameters are *w* = 50 nm, *g* = 20 nm, and *t* = 10 nm.

**Figure 2:**
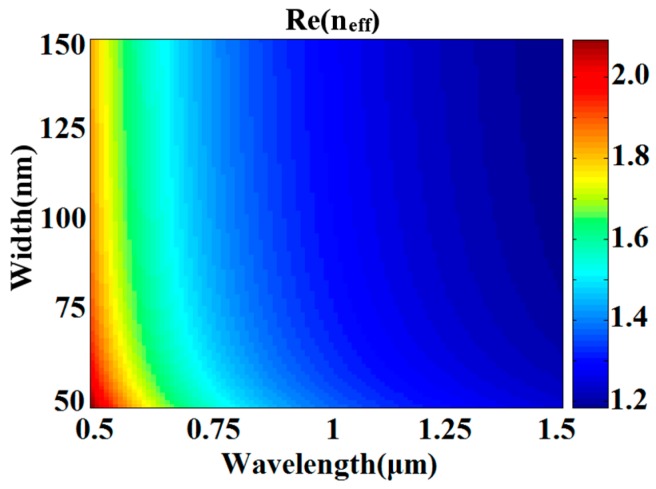
Real part of the effective refractive index *n_eff_* versus the incident wavelength *λ* and the width *w* in MIM waveguide.

Multiple ring resonators are widely used in optical filtering. Here we used two ring resonators to investigate the design consideration about the optical filter. The proposed structure, shown schematically in Figure 1, consists of two ring resonators and two waveguides with a baffle (the blue area). The system is a two-dimension model. The FEM with COMSOL Multiphysics is employed to realize our simulations. The calculated area is divided by Yee’s mesh with a size of 2 nm. The FEM with scattering boundary condition is employed to investigate the transmission characteristics of the structure. Two MIM waveguides are marked as I and II. Port 1 and Port 2 are input and output ports, respectively. We assumed the media inside the rings and waveguides to be air (the white area in Figure 1). The widths of the waveguides and the rings, both *w*, are fixed at 50 nm. Since the widths *w* is much smaller than the wavelength of the incident light, only the fundamental plasmonic mode TM_0_ could exist in the structure. The TM_0_-polarized plane wave launched to the left waveguide is used for exciting SPP waves, which is indicated by the arrow in Figure 1. The outer (inner) radii of the two rings are *r*_1_ (*r*_2_) and *r*_1_’ (*r*_2_’), respectively. Meanwhile, we defined *r* = (*r*_1_ + *r*_2_)/2 and *r*’ = (*r*_1_’ + *r*_2_’)/2 as the radii of two ring resonators; *t* is the coupling distance between the waveguide and the ring; *g* is the thickness of the baffle. The metal is set as silver, whose frequency-dependent dielectric constant is given by the well-known Drude model [5]:

(1) $$\varepsilon_{m}\left(\omega \right)=\varepsilon_{\infty }-\frac{\omega_{p}^{2}}{\omega \left(\omega +i\gamma \right)}$$

where *ε_∞_* = 3.7 is the dielectric constant at the infinite frequency, *γ* = 2.73 × 10^13^ Hz is the electron collision frequency, *ω_p_* = 1.38 × 10^16^ Hz is the bulk plasma frequency, and *ω* stands for the angular frequency of the incident electromagnetic radiation. The propagation constant *β* of SPPs is determined by the following equation [5]:

(2) $$\tanh\left(\frac{w\sqrt{\beta^{2}-k_{0}^{2}\varepsilon_{i}}}{2}\right)=\frac{-\varepsilon_{i}\sqrt{\beta^{2}-k_{0}^{2}\varepsilon_{m}\left(\omega \right)}}{\varepsilon_{m}\left(\omega \right)\sqrt{\beta^{2}-k_{0}^{2}\varepsilon_{i}}}$$

where *ε_m_* and *ε_i_* are the dielectric constants of the silver and air, respectively. *k*_0_ is the wave vector of light in vacuum. The effective refractive index follows *n_eff_* = *β/k*_0_. The real part of *n_eff_* as a function of *w* and *λ* is shown in Figure 2. For a fixed wavelength, *Re* (*n_eff_*) gradually grows as wavelength *λ* increases. Theoretically, the resonant wavelength of the ring resonator can be derived from the equation:

(3) $$\frac{J_{n\prime }^{\prime }\left(kr_{1}\right)}{J_{n\prime }^{\prime }\left(kr_{2}\right)}=\frac{N_{n\prime }^{\prime }\left(kr_{1}\right)}{N_{n\prime }^{\prime }\left(kr_{2}\right)}$$

where *k* = *ω(ε*_0_*ε_γ_μ*_0_*)*^1/2^, and *ε*_0_ and *μ*_0_ are the dielectric constant and permeability in vacuum, respectively. *ε_γ_* = *(n_eff_)*^2^/*μ*_0_ is the frequency-dependent effective relative permittivity. $J_{n\prime }$ is the Bessel function of the first kind with order *n*’, and $N_{n\prime }$ is the Bessel function of the second kind with order *n*’. $J_{n\prime }^{\prime }$ and $N_{n\prime }^{\prime }$ are the derivatives of the Bessel functions to the argument *kr*. The resonant wavelength also satisfies the simple relation *Re* (*n_eff_*) *L* = *mλ*, where *m* is the resonant mode number, a positive integer.

## 3. Results and Discussion

**Figure 3:**
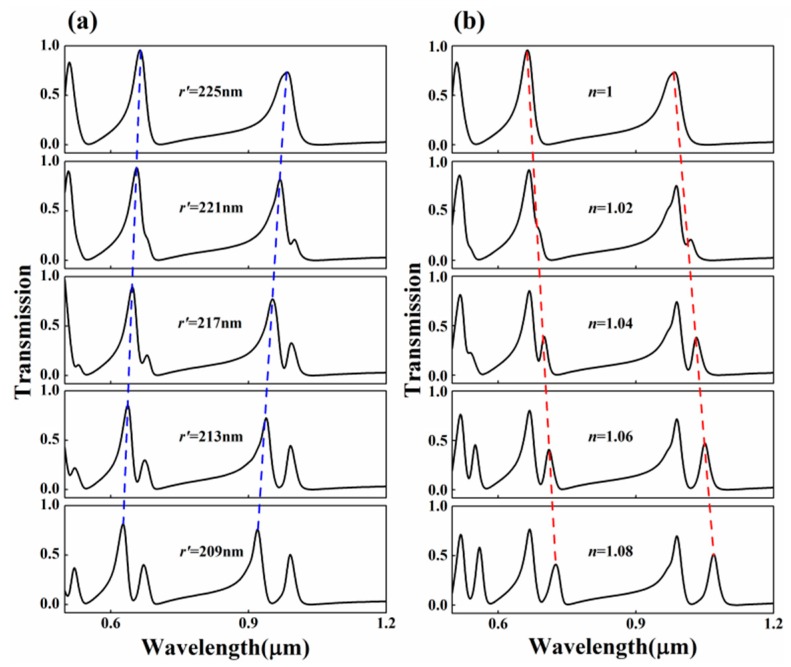
Transmission spectra of two shunt-wound rings system (**a**) for different radius *r*’ of the lower ring; (**b**) for different refractive index *n* of the lower ring.

**Figure 4:**
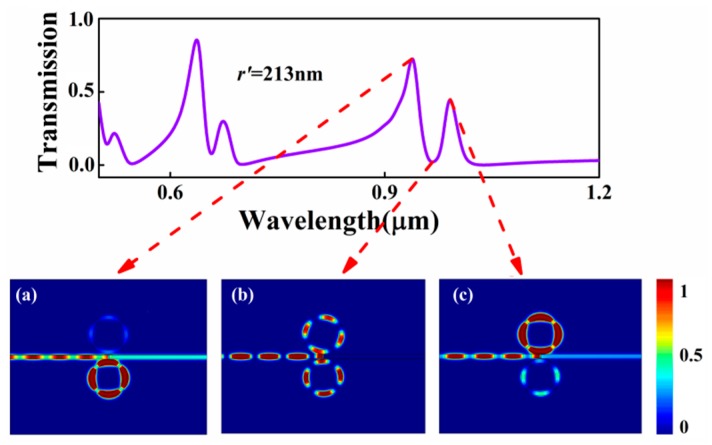
Transmission spectrum of the MIM waveguide with two shunt-wound ring resonators when *r*’ = 213 nm; the contour profiles of magnetic intensity distributions |*H*_z_|^2^ of the device at (**a**) *λ* = 940 nm; (**b**) *λ* = 967 nm; (**c**) *λ* = 991 nm.

Firstly, we focused on this two shunt-wound rings system, shown in Figure 1. The baffle is assumed to be silver. In this case, the two rings cannot interact with each other. Therefore, one portion of SPPs coupled to the upper ring, the other to the lower ring, both of them will interference with the third portion, which pass through the baffle. At first, the influence of the radius *r*’ of the lower ring was discussed. We set *r*’ as from 225 nm to 209 nm at the step of 4 nm. Other geometric parameters were chosen as follows: *r* = 225 nm, *t* = 10 nm, and *g* = 20 nm. The simulation results are plotted in Figure 3a. Figure 3a clearly shows that, when *r* = *r*’ = 225 nm, sharp and asymmetrical spectral profiles, which are regarded as Fano resonances [32,33], can be obtained. Here, Fano resonances result from the interference of the broad spectrum and the discrete resonance, which are caused by the baffle and the ring cavities, respectively. As *r*’ decreases, it is obviously observed that the transmittance peak of each mode splits into two and the dips at different modes are becoming more apparent. Moreover, the left peaks of each mode exhibit a blue shift (denoted by the blue dotted line) with *r*’ decreasing, and the right keep almost unchanged because *r* is fixed to 225 nm. It goes without saying that the left peaks are determined by the lower ring. Figure 4 shows the corresponding field distributions of |*H*_z_|^2^ when *r* = 225 nm and *r*’ = 213 nm. It is observed that the energy of the resonant peaks is confined in only one cavity, the energy of the dip in two rings. These behaviors of the two resonances accord well with the analysis.

In a similar way, we provided another way to control the transmission windows by changing the refractive index *n* of the lower ring. The refractive index *n* of the lower ring was set from 1 to 1.08 with a step of 0.02, and other parameters were kept the same as the above. We plotted the simulated results in Figure 3b. As *n* grows, the dips are becoming more obvious and the right peaks of each mode show a little red-shift (denoted by the read dotted line) in the spectra. Similarly, the shifting effect is more apparent and the width of dips is wider at low resonant modes. From the aforementioned analysis, two methods were found to achieve on-to-off PIA response in a two shunt-wound rings filter system.

In addition, the influences of baffle dimension and position on the transmission spectrum were investigated. The results showed that, as *g* grows, the transmission peaks gradually lower; when the baffle shift to the right or the left is less than 150 nm, the transmission peaks do not show a shift in the x-axis; when the shifting distance is more than 150 nm, little energy is transmitted.

**Figure 5:**
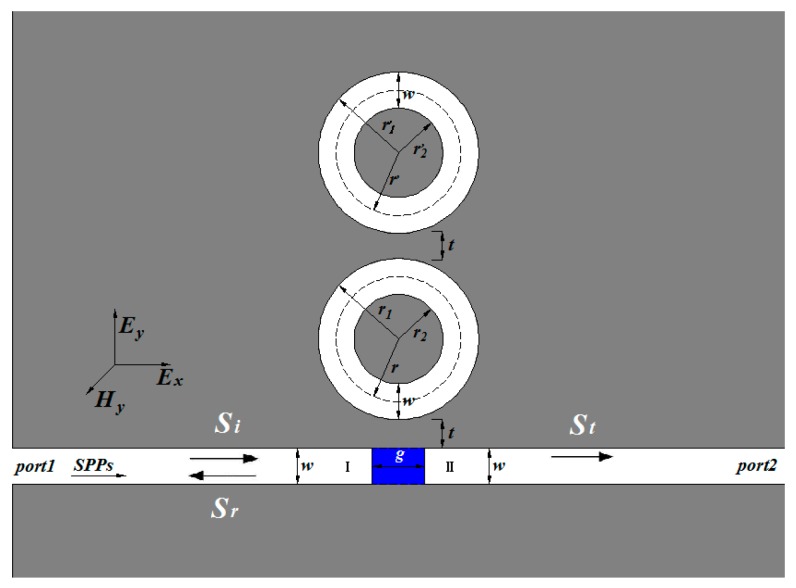
Schematic of the structure composed of two series-wound ring resonators with two waveguides.

**Figure 6:**
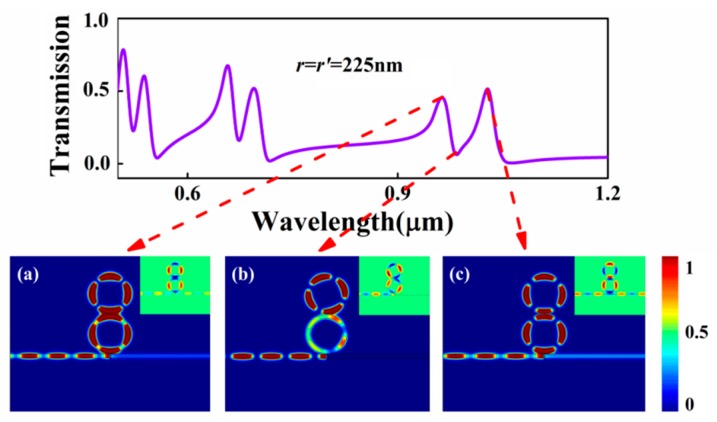
Transmission spectrum of the MIM waveguide with two series-wound ring resonators when *r* = *r*’ = 225 nm; the contour profiles of magnetic intensity distributions |*H*_z_|^2^ of the device at (**a**) *λ* = 961 nm; (**b**) *λ* = 986 nm; (**c**) *λ* = 1027 nm. The insets denote the corresponding *H*_z_ distribution of SPPs in the PIT systems.

Successively, in addition to the above case, the structure proposed in Figure 1 can be also extended to another situation, the MIM bus waveguide side-coupled two series-wound rings, shown in Figure 5. The separation between two rings is set as 10 nm and the radii of the two rings are all set as 225 nm; other parameters are kept the same as above. Due to the side-coupled arrangements, the bottom ring cavity couples strongly to the bus waveguide, the so-called bright mode; the upper ring cavity cannot directly couple to the bus waveguide, known as the dark mode. On account of the greatly enhanced near-field interferences between two rings, the dark mode is excited through tunneling coupling with the bright mode. As a result, PIA transmission appears at the pass band of the structure without the upper ring cavity, with two new resonant peaks generated as shown in Figure 5. The corresponding magnetic intensity distributions *|H*_z_*|*^2^ at the two peaks and the one dip for the second-order mode are displayed in Figure 6a–c. Obviously, unlike shunt-wound rings system, for transmission peaks, the bottom ring is directly excited by the incident SPPs, and the upper ring is also enhanced thanks to the coupling with the bottom ring resonator. The in-phase coupling resonance occurs at the two rings with the wavelength of *λ* = 961 nm, shown in the inset of Figure 6a, whereas the out-phase forms at the wavelength of *λ* = 1027 nm shown in the inset of Figure 6c. The incident SPP power can be coupled into the right waveguide by the bottom ring, leading to the newly appeared transmission peaks. For the dip, however, the dark resonator is efficiently activated, whereas the bright resonator is extraordinarily suppressed due to the strong destructive interference. Almost all SPP power is absorbed in the upper ring cavity, and little SPP power is coupled into the right MIM waveguide output shown in Figure 6b, which is direct evidence of the remarkable absorption window in the intrinsic peak point. Hence, the introduced upper ring cavity can store all SPP power and achieve PIA response.

On the other hand, the transmission properties can be analyzed by the temporal coupled mode theory (CMT) [34,35]. As shown in Figure 5, the amplitudes of the incident, reflected, and total-transmitted SPP waves are denoted by *S_i_*, *S_r_*, and *S_t_*, respectively, and are normalized to the power in the modes. *Q_r_* is cavity quality factors related to intrinsic loss in the dual rings; *Q_w_* is the quality factor related to the coupling loss between waveguides and the bottom ring; *Q_c_* is the quality factor related to the coupling loss between two rings. The time evolution normalized amplitude *a*_1_ of the bottom ring and *a*_2_ of the upper ring can be described from the CMT:

(4) $$\frac{da_{1}}{dt}=\left(j\omega_{0}-\frac{\omega_{0}}{2Q_{r}}-\frac{\omega_{0}}{2Q_{w}}\right)a_{1}+\sqrt{\frac{\omega_{0}}{Q_{w}}}S_{i}-j\frac{\omega_{0}}{2Q_{c}}a_{2}$$

(5) $$\frac{da_{2}}{dt}=\left(j\omega_{0}-\frac{\omega_{0}}{2Q_{r}}\right)a_{2}-j\frac{\omega_{0}}{2Q_{c}}a_{1}$$

where *ω*_0_ is the same resonant frequency of the dual rings. According to energy conservation, the amplitude of the input and the output lights in the waveguide should satisfy the following relationships:

(6) $$S_{r}=-S_{i}+j\sqrt{\frac{\omega_{0}}{Q_{w}}}a_{1}$$

(7) $$S_{t}=j\sqrt{\frac{\omega_{0}}{Q_{w}}}a_{1}.$$

Therefore, the transmission coefficient *T* can be obtained as follows:

(8) $$T={\left|\frac{S_{t}}{S_{i}}\right|}^{2}={\left|\frac{2}{Q_{w}}\frac{j2\left(\frac{\omega -\omega_{0}}{\omega_{0}}\right)+\frac{1}{Q_{r}}}{{\left(j2\left(\frac{\omega -\omega_{0}}{\omega_{0}}\right)+\frac{1}{Q_{r}}+\frac{1}{2Q_{w}}\right)}^{2}+{\left(\frac{1}{Q_{c}}\right)}^{2}-{\left(\frac{1}{2Q_{w}}\right)}^{2}}\right|}^{2}.$$

**Figure 7:**
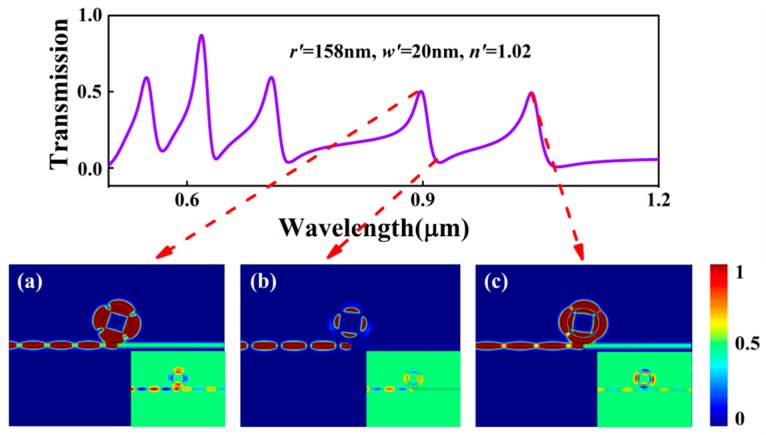
Transmission spectrum of the MIM waveguide with two concentric rings resonators when *r*’ = 158 nm, *w*’ = 20 nm, and *n*’ = 1.02; the contour profiles of magnetic intensity distributions *|H*_z_*|*^2^ of the device at (**a**) *λ* = 898 nm; (**b**) *λ* = 921 nm; (**c**) *λ* = 1035 nm. The insets denote the corresponding *H*_z_ distribution of SPPs in the concentric rings system.

Finally, the two concentric rings system was investigated. As shown in Figure 7, the radius *r*’, width *w*’, and refractive index *n*’ of the inner ring are set at 158 nm, 20 nm, and 1.02, respectively; other parameters are kept the same as the above. The transmission spectrum was calculated and is shown in Figure 7. According to the above theoretical analysis results, for coupling directly to the bus waveguide, the outer ring is called the bright mode, and on the contrary, the inner ring is called the dark mode. Similarly, on account of the greatly enhanced near-field interferences between two rings, it is obvious that two resonant peaks appear in each mode in the transmission spectrum. In order to find out the underlying physics of the resonant peaks in the transmission spectrum, the magnetic intensity distributions *|H*_z_*|*^2^ at the two peaks and one dip for the second-order mode are displayed in Figure 7a–c. The same as series-wound rings system, for transmission peaks, the outer ring is directly excited by the incident SPPs and the inner ring couples with the outer ring. The in-phase and out-phase coupling resonance occurs between the two rings at *λ* = 898 nm and *λ* = 1035 nm, respectively, shown in the insets of Figure 7a,c. However, for the dip, due to the strong destructive interference, the bright resonator is suppressed and the dark resonator is efficiently activated. Almost all SPP energy is absorbed in the inner ring cavity, and little SPP energy is coupled into the right MIM waveguide output shown in Figure 7b. These results provide a theoretical basis for designing highly integrated optical devices.

## 4. Conclusions

In summary, we studied the transmission characteristics of a wavelength band-pass filter consisting of dual side-coupled circular ring resonators and two waveguides with a baffle. For the two shunt-wound rings system, we provided two approaches to control the transmission spectra. An on-to-off PIA optical response could be achieved by adjusting the radius or the refractive index of the lower ring. We found the shifting effect is more apparent and the width of the absorption dips is wider at low resonant modes. For the series-wound rings system and the concentric rings system, the corresponding transmission spectra and the magnetic intensity distributions *|H*_z_*|*^2^ at special wavelengths were investigated. Compared with the shunt-wound rings system, it is difficult to achieve the on-to-off PIA optical response by adjusting the structural parameters of these two systems although there are PIA windows in the transmission spectra. The analyses make these kinds of experiments easier and pave the way for actively tunable sensoring applications.
